# Accelerated repetitive transcranial magnetic stimulation (ATMS) vs standard repetitive transcranial magnetic stimulation (RTMS) in the treatment of major depressive episodes. preliminary data of a randomized, single-blind, controlled trial

**DOI:** 10.1192/j.eurpsy.2023.316

**Published:** 2023-07-19

**Authors:** M. Prato, N. Ragone, C. Passani, V. Cardaci, F. Seghi, B. Barbini, C. Colombo

**Affiliations:** 1 Mood Disorder Unit, IRCCS Ospedale San Raffaele, Ville Turro, Milan, Italy; 2 Vita-Salute San Raffaele University, Milan, Italy

## Abstract

**Introduction:**

Major Depressive Disorder is a frequent and disabling condition. More than 20% of patients do not respond to pharmacotherapy alone, so there is the need to find alternative strategies in order to potentiate the drugs. Therapeutic alternatives include repetitive Transcranial Magnetic Stimulation (rTMS), which has shown an antidepressant effect in the last decades.

**Objectives:**

Comparison of the efficacy of accelerated repetitive Transcranial Magnetic Stimulation (aTMS) treatment (4 sessions/day for 5 days) with the standard rTMS protocol treatment (1 session/day for 4 weeks), using the FDA-approved parameters.

**Methods:**

33 patients affected by Major Depressive Episodes treated with either Fluvoxamine or Venlafaxine were enrolled. Patients were randomly assigned to the two protocol groups: standard rTMS protocol (15 patients) and aTMS protocol (18 patients). In the standard protocol, patients received 1 rTMS session/day for 4 weeks, while in the aTMS protocol they received 4 rTMS sessions/day for 5 days. Symptomatological improvement was evaluated through MADRS, BDI-II and SSI rating scales administered on day: 0, 1, 2, 3, 4, 5, 14, 21, 28, 56. The study is single-blind, since the clinical rater was unaware of the treatment protocol group. Response and remission rates were calculated, defined respectively as a reduction ≥50% in the MADRS score and a MADRS score <10.

**Results:**

The analysis was carried out on 32 patients (18 in the aTMS group and 14 in the rTMS group). ANOVA for repeated measures shows a statistically significant difference in the MADRS scores on day 5 (*p*=0.001) and on day 56 (*p*=0.037). Regarding the BDI-II evaluation, the differences were not fully statistically significant on day 5 and not significant on day 56. No statistically significant differences between the two protocols were observed in the SSI assessment. The aTMS and rTMS response rates were respectively 84.6% vs 45.5% on day 28 (*p*=0.043) and 92.3% vs 45.5% on day 56 (*p*=0.012). The aTMS and rTMS group remission rates were respectively 76.9% vs 18.2% on day 28 (*p*=0.004) and 69.2% vs 36.4% on day 56 (*p*=0.107). Concerning side effects, no statistically significant differences were observed between the two groups.

**Conclusions:**

Treatment with aTMS seems faster and more effective than treatment with standard rTMS in improving the clinical condition in patients with Major Depressive Episodes, allowing to treat patients in just 5 days instead of 4-6 weeks, without impacting on side effects and tolerability.

**Disclosure of Interest:**

None Declared

